# Metformin decreases LPS-induced inflammatory response in rabbit annulus fibrosus stem/progenitor cells by blocking HMGB1 release

**DOI:** 10.18632/aging.102453

**Published:** 2019-11-26

**Authors:** Yingchao Han, Feng Yuan, Chao Deng, Fan He, Yan Zhang, Hongxing Shen, Zhi Chen, Lie Qian

**Affiliations:** 1 Department of Spine Surgery, Renji Hospital, School of Medicine, Shanghai Jiao Tong University, Shanghai 200127, China; 2 Department of Sports Medicine, Shanghai Sixth People’s Hospital, School of Medicine, Shanghai Jiao Tong University, Shanghai 200233, China; 3 Department of Spinal Surgery, Shanghai East Hospital, Tongji University, School of Medicine, Shanghai 200120, China

**Keywords:** intervertebral disc degeneration, annulus fibrosis stem cells, cell senescence, HMGB1, metformin

## Abstract

The present study aimed to investigate the mechanism of intervertebral disc degeneration (IVDD) and identify an efficient treatment for low back pain. Rabbit annulus fibrosus stem cells (AFSCs) were treated with metformin and lipopolysaccharide (LPS). The results indicated that LPS induced HMGB1 release from the nuclei of AFSCs and caused cell senescence in a concentration-dependent manner. The production of PGE2 and HMGB1 was increased in the medium of the LPS-treated AFSCs. Certain inflammation-associated genes (*IL-β1*, *IL-6*, *COX-2* and *TNF-*α) and proteins (IL-β1, COX-2 and TNF-α) and specific catabolic genes (*MMP-3* and *MMP-13*) exhibited increased expression in LPS-treated AFSCs. However, the expression levels of other anabolic genes, such as *collagen I* and *collagen II* were decreased in LPS-treated AFSCs. Following addition of metformin to LPS-containing medium, HMGB1 was retained in the nuclei of AFSCs and the production of PGE2 and HMGB1 was reduced. The expression levels of the catabolic genes and proteins were decreased and those of the anabolic genes were increased. The findings indicated that metformin exerted an anti-inflammatory effect by blocking the HMGB1 translocation and by inhibiting catabolic production and cell senescence in AFSCs. Therefore, metformin may be used as an efficient treatment for the disc degenerative disease.

## INTRODUCTION

Low back pain is a very common disc disease with a tremendous socio-economic impact. More than 70% of the worldwide population has encountered low back pain during various stages of life [1]. The disease severity is more common in aging populations [1]. Although novel strategies are currently being developed for preventing degeneration or promoting regeneration of the intervertebral disc, they do not lead to complete remission of the intervertebral disc disease and its treatment remains a major challenge for both clinical doctors and orthopedic researchers [2]. To date, an efficient treatment for low back pain has not been developed, while the etiology of this disease is largely unknown. The intervertebral disc degeneration (IVDD) is a widely accepted cause of low back pain [2]. It is believed that certain inflammatory mediators are associated with herniated and degenerated intervertebral disc diseases. *In vitro* studies have shown that IL-6, IL-8 and PGE2 levels were increased in human intervertebral disc tissues following lipopolysaccharide (LPS) stimulation [3]. The findings indicated that pro-inflammatory cytokine release, increased matrix catabolism, induction of cell apoptosis and cell senescence were biological processes involved in the pathogenesis of IVDD [2, 4, 5]. However, the precise cellular and molecular mechanism of IVDD is not clear [2, 4, 5].

It has been shown that stem cells play a key role in tissue regeneration and degeneration. Disc stem/progenitor cells have been isolated from human and animal spinal disc tissues [6, 7]. Disc degeneration is classified as a disease of aging, characterized by loss of viable cells and an increase in cell senescence [8]. It is well known that stem cells have a multi-differentiation potential, which allows them to differentiate into various cell types, such as adipocytes, chondrocytes and osteocytes. The discs from patients with spinal deformities exhibit ectopic calcification in the cartilage end plate and in the disc itself [9]. It has been reported that lumbar disc degeneration is associated with modic type endplate changes and high paraspinal fat content [10]. However, the exact cause of degeneration and senescence of disc stem cells is largely unknown.

High mobility group box 1 (HMGB1) is a nuclear protein that binds to DNA and acts as a co-factor for gene transcription [11]. Generally, the resting state form of the HMGB1 protein exists in the nuclei of the majority of cells and regulates DNA stability and gene expression. However, the activated form of HMGB1 can be released from the nuclei of the stimulated, injured and necrotic cells into the extracellular space [12]. Once released, the extracellular HMGB1 plays an important role in cell proliferation and migration, as well as in the development and maintenance of the inflammatory response [13–15]. It has been shown that the released HMGB1 protein enhances the production of PGE2, IL-1β, IL-6 and TNF-α in the extracellular matrix of the cells [16, 17].

The effect of extracellular HMGB1 in the pathogenic process of several diseases, such as cancer, stroke, endotoxemia, and joint disorders has been studied [18–20]. However, a limited number of studies have focused on the regulatory role of HMGB1 in the inflammatory response of IVD cells.

Metformin is a widely used drug for type 2 diabetes [21]. Recent studies have shown that metformin can serve as a potential drug to treat inflammation-related disorders [22, 23]. However, the mechanism of metformin anti-inflammatory action is not clearly understood [24]. The present study aimed to determine whether metformin could regulate inflammation by inhibiting the release of HMGB1 in LPS-treated IVD cells using an *in vitro* rabbit annulus fibrosus (AF) stem cell model.

## RESULTS

### Isolation and identification of rabbit AFSCs

**Figure 1:**
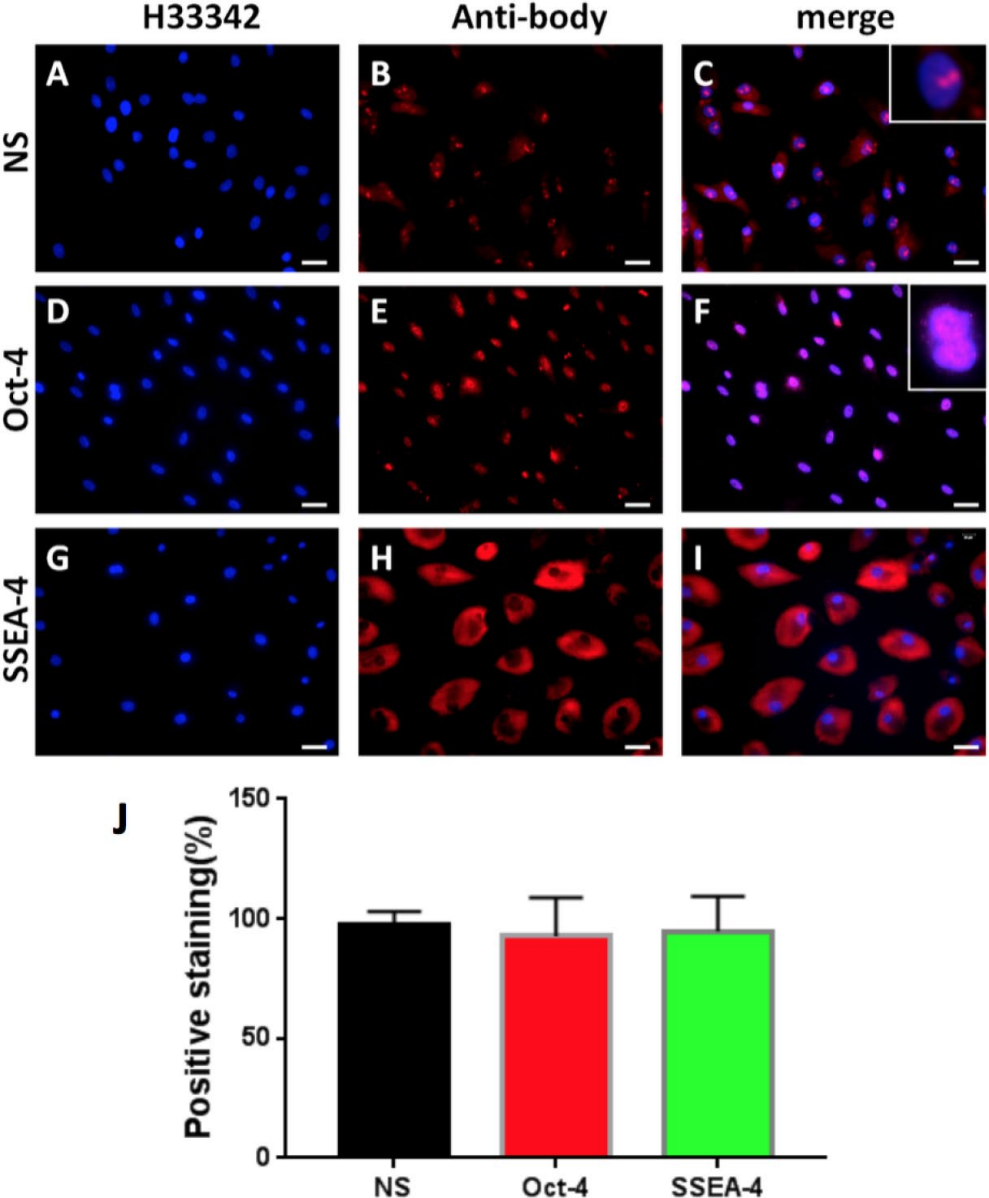
**Stem cell marker expression of rabbit AF cells tested by immunostaining.** (**A**–**C**) nucleostemin testing; (**D**–**F**) Oct-4 testing; (**G**–**I**) SSEA-4 testing. (**A**, **D**, **G**) the cells were stained with H33342; (**B**, **E**, **L**) the cells were stained with specific antibodies; (**C**, **F**, **I**) the merged images of the images of **A**, **D**, **G** and the images of **B**, **E**, **L**. The insets showed enlarged views of expressed nucleostemin (**C**) and Oct-4 (**F**). (**J**) Semi-quantification of the expression of three stem markers by immunostaining. The results indicated that more than 92% of the cells isolated from rabbit AF tissues were stem cells. Bars = 100 μm.

In order to study the cellular and molecular pathway of disc degeneration, stem cells were initially isolated from rabbit AF tissues (AFSCs) and the stemness of these AFSCs was identified by three stem cell markers, namely octamer-binding transcription factor-4 (Oct-4), stage-specific embryonic antigen-4 (SSEA-4) and nucleostemin (NS). Immunostaining results indicated that more than 92% of AFSCs were positively stained with all three stem cell markers (Figure 1), suggesting these AFSCs could be used for the following experiments.

### The effect of metformin and LPS on cell morphology and proliferation

**Figure 2:**
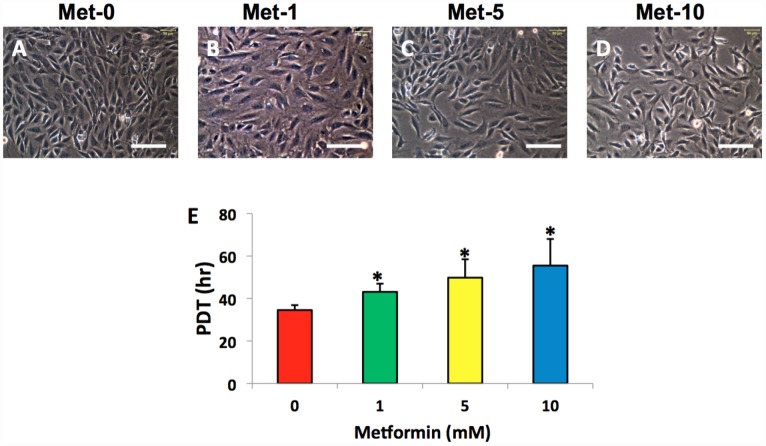
**Metformin effect on the proliferation of rabbit AF cells cultured for 7 days.** Metformin didn’t induce the morphological change significantly in rabbit AF cells during the culture (**A**–**D**), while the proliferation of the rabbit AF cells was decreased at a metformin concentration dependent manner as evidenced by population doubling time (**E**). *p<0.05 compared to the control group (Met-0, Met-1, Met-5, and Met-10 represent that the concentrations of metformin in the medium are 0, 1, 5, 10 mM, respectively). White bars = 100 μm.

**Figure 3:**
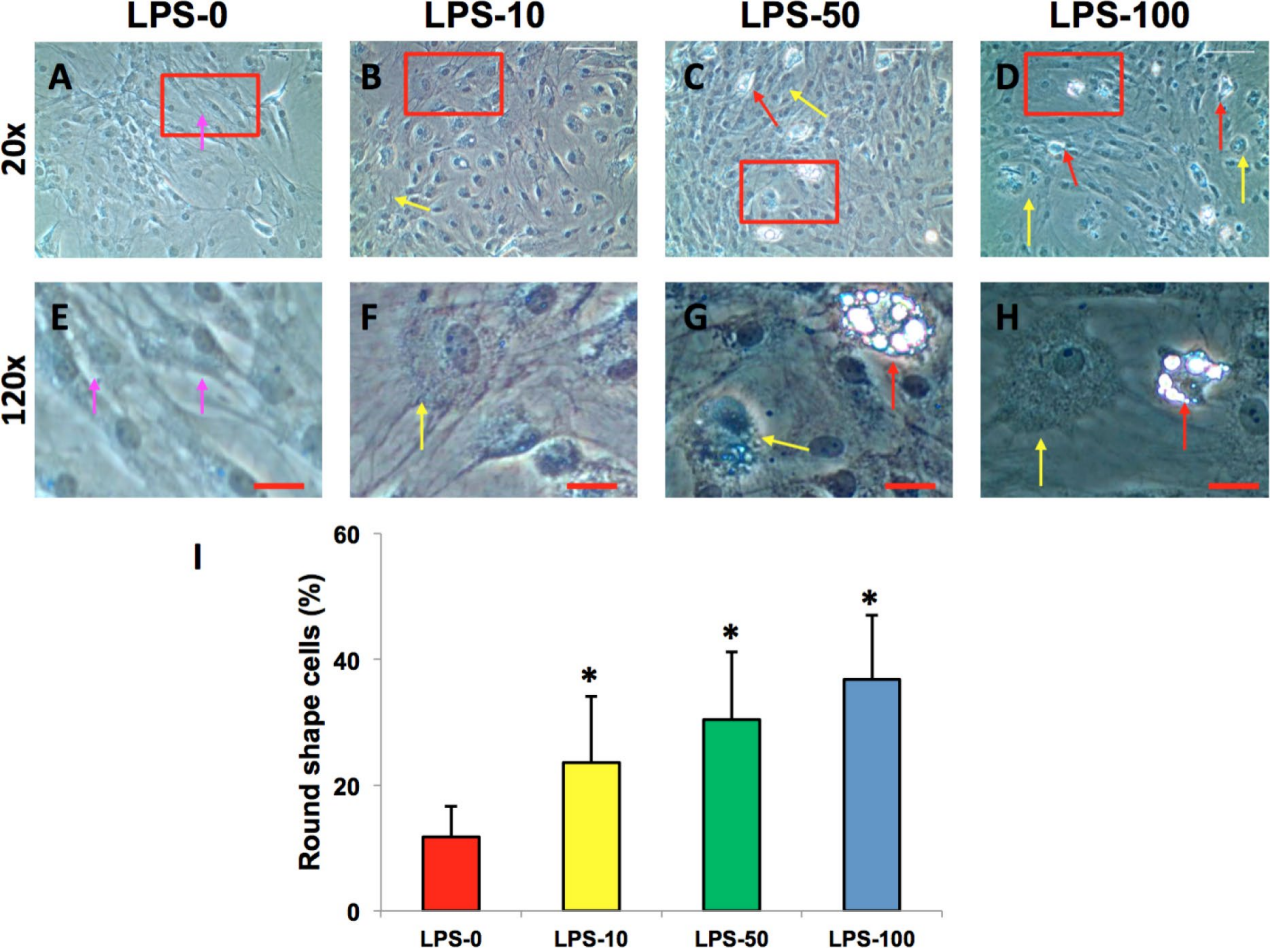
**LPS effect on the proliferation and differentiation of rabbit AF cells cultured for 7 days.** LPS not only decreased the proliferation of rabbit AF cells, but also induced significantly morphological change in rabbit AF cells during the culture (**A**–**H**). The results showed that the cell number was decreased with increasing the concentration of LPS. Many cells have changed their shape from spindle shape to round shape (yellow arrows in **B**–**D**, **F**–**H**). Some adipocyte-like cells were also found in the LPS-treated groups (red arrows in **C**, **D**, **G**, **H**). Semi-quantification indicated that the round shape cells were increased at a LPS concentration dependent manner (**I**). The images of **E**–**H** were the enlarged images of the box areas in the images of **A**–**D**. *p<0.05 compared to the control group. White bars = 200 μm, red bars = 50 μm.

The AFSCs isolated from rabbit AF tissues were treated with various concentrations of metformin (0–10 mM) for 7 days. Although metformin did not change the morphology of AFSCs (Figure 2A–2D), it decreased the proliferation of rabbit AF cells at a concentration dependent manner (Figure 2E). However, the morphology of rabbit AF cells was altered from spindle-like (pink arrows in Figure 3A, 3E) to epithelial-like cells following LPS treatment (yellow arrows in Figure 3B–3D, 3F–3H). Semi-quantification indicated that the number of round-shape cells was increased following an increase in the concentration of LPS (Figure 3I). Certain adipocyte-like cells were also evident in LPS-treated cells (red arrows in Figure 3C, 3D, 3G, 3H).

### Metformin inhibits LPS-induced HMGB1 release from the nuclei of AFSCs

**Figure 4:**
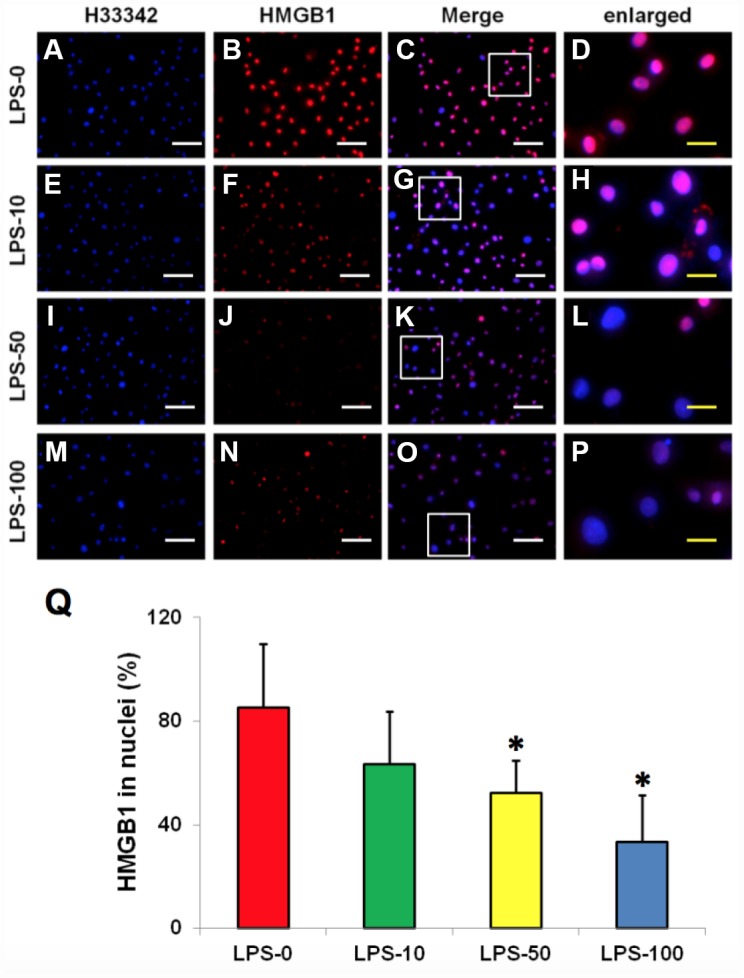
**LPS effect on HMGB1 releasing from the nuclei to the cytoplasma of the rabbit AF cells cultured for 7 days.** (**A**–**D**) normal rabbit AF cells (LPS-0). (**E**–**H**) the rabbit AF cells were treated with 10 ng of LPS (LPS-10). (**I**–**L**) the rabbit AF cells were treated with 50 ng of LPS (LPS-50). (**M**–**P**) the rabbit AF cells were treated with 100 ng of LPS (LPS-100). (**Q**) semi-quantification of positive stained HMGB1 in the nuclei of the cells. More than 95% of the nuclei of the normal cells were positively stained with HMGB1 (**B**–**D**, **Q**). LPS induced HMGB 1 releasing from the nucleus of rabbit AF cells to the cytoplasma (**F**–**H**, **J**–**L**, **N**–**P**). Semi-quantification indicated that the concentration of HMGB1 in the cytoplasma increased with increasing of the concentration of LPS (**Q**). (**A**, **E**, **I**, **M**) H33342 staining. (**B**, **F**, **J**, **N**) anti-HMGB1 antibody staining*p<0.05 compared to the control group. (**C**, **G**, **K**, **O**) merged images of H33342 stained images and anti-HMGB1 antibody stained images. (**D**, **H**, **L**, **P**) enlarged images of the box areas in the images of (**C**, **G**, **K**, **O**). White bars = 100 μm, yellow bars = 25 μm.

**Figure 5:**
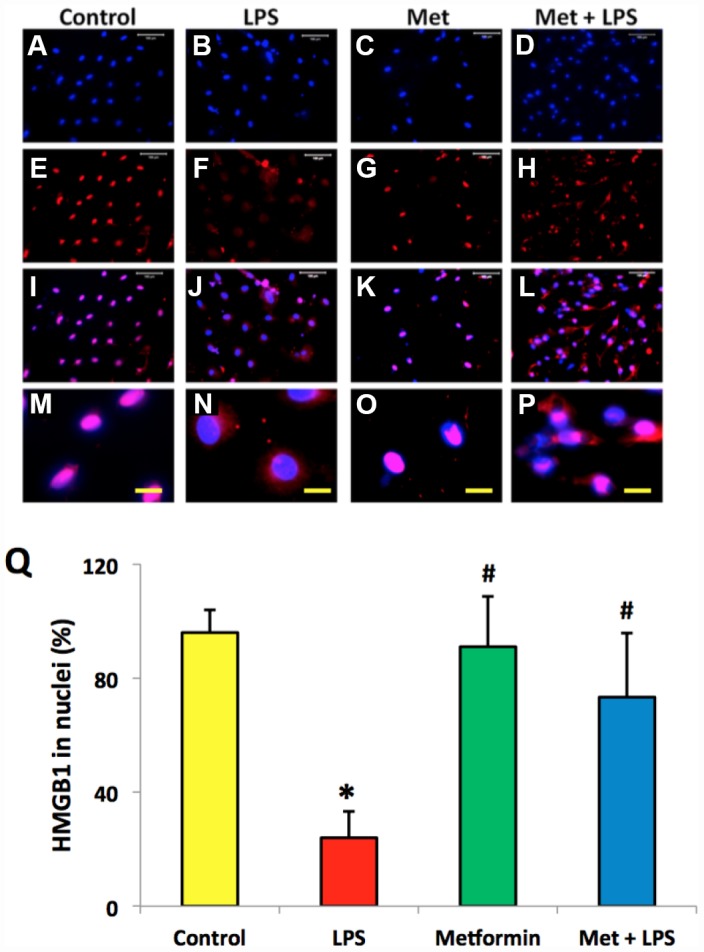
**Metformin inhibited the relocation of HMGB1 in rabbit AF cells induced by LPS.** (**A**–**D**) H33342 staining. (**E**–**H**) anti-HMGB1 antibody staining. (**I**–**L**) merged images of the H33342 stained images (**A**–**D**) and anti-HMGB1 antibody stained images (**E**–**H**). (**M**–**P**) the enlarged images of the box areas of the images of (**E**–**H**, **Q**) semi-quantification of positive stained HMGB1 in the nuclei of the cells. More than 95% of the nuclei of the normal cells were positively stained with HMGB1 (**I**, **M**, **Q**). LPS induced HMGB 1 releasing from the nucleus of rabbit AF cells to the cytoplasma (**J**, **N**, **Q**). Metformin blocked the relocation of HMGB1 as evidenced by the concentration of HMGB1 in the nuclei (**K**, **O**, **Q**). *p<0.05 compared to the control group. ^#^p<0.05 compared to the cells treated with LPS. White bars = 100 μm, yellow bars = 25 μm.

Immunostaining indicated that more than 85% of HMGB1 was localized in the nuclei of normal rabbit AF cells (Figure 4A–4D, 4Q). However, LPS induced HMGB1 release from the nuclei of rabbit AF cells to the cytoplasm at a concentration dependent manner (Figure 4E–4Q). Less than 20% of HMGB1 expression was noted in the nuclei of rabbit AF cells following treatment with 100 ng/ml LPS (Figure 4N–4Q). Metformin inhibited the translocation of HMGB1 in rabbit AF cells as demonstrated by the high percentage of HMGB1 expression noted in the nuclei of rabbit AF cells treated either with metformin alone (Figure 5C, 5G, 5K, 5O, 5Q), or with metformin and LPS (Figure 5D, 5H, 5L, 5P, 5Q).

### Metformin inhibits LPS- induced cell senescence of AFSCs

**Figure 6:**
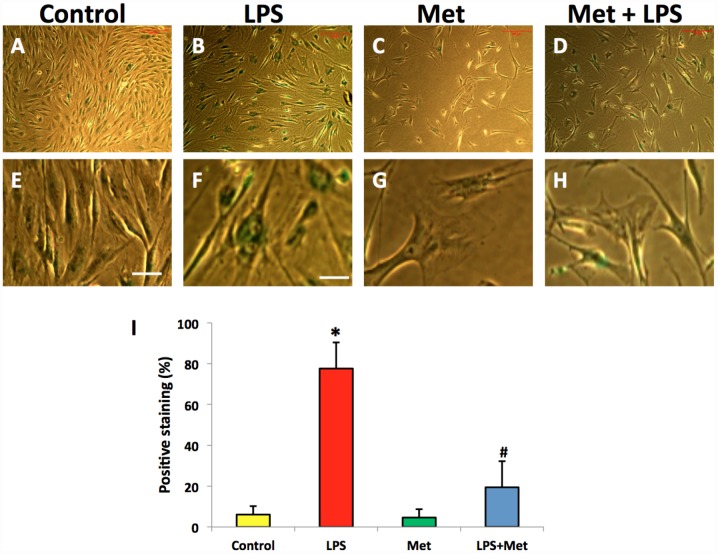
**Metformin inhibited cell senescence of rabbit AF cells induced by LPS tested by b-gal staining.** (**A**, **E**) the cells were cultured with normal medium (control). (**B**, **F**) the cells were cultured with 100 ng/ml of LPS-containing medium (LPS). (**C**, **G**) the cells were cultured with 1 mM of metformin-containing medium (Met). (**D**, **H**) the cells were cultured with 100 ng/ml of LPS and 1 mM of metformin (LPS+Met). The images of (**E**–**H**) were enlarged images of the box areas of the images of (**A**–**D**) LPS induced more than 77% of rabbit AF cells to senescence as evidenced by b-gal staining (green cells in **B**, **F**). Metformin inhibited cell senescence induced by LPS (**D**, **H**), (**I**) semi-quantification of positive stained cells by b-gal. *p<0.05 compared to the control group. ^#^p<0.05 compared to the cells treated with LPS. White bars = 100 μm, yellow bars = 25 μm.

SA-β-gal staining indicated that LPS (100 ng/ml) induced senescence of more than 75% of the total population of AFSC cells (Figure 6B, 6F, 6I). Moreover, metformin could inhibit LPS-induced cell senescence (Figure 6D, 6H).

### Metformin inhibits LPS-induced inflammation in AFSCs

The effects of metformin and LPS treatment on the inflammatory response of rabbit AF cells were further investigated by qRT-PCR on AF stem cells (Figure 6). LPS treatment increased inflammatory gene expression levels in AF cells (Figure 6). The expression of TNF-α was 7 fold-higher in AF stem cells treated with LPS (Figure 6A). Furthermore, the expression levels of IL-β1 (Figure 6B), IL-6 (Figure 6C) and COX-2 (Figure 6D) were also increased in LPS-treated rabbit AF cells.

**Figure 7:**
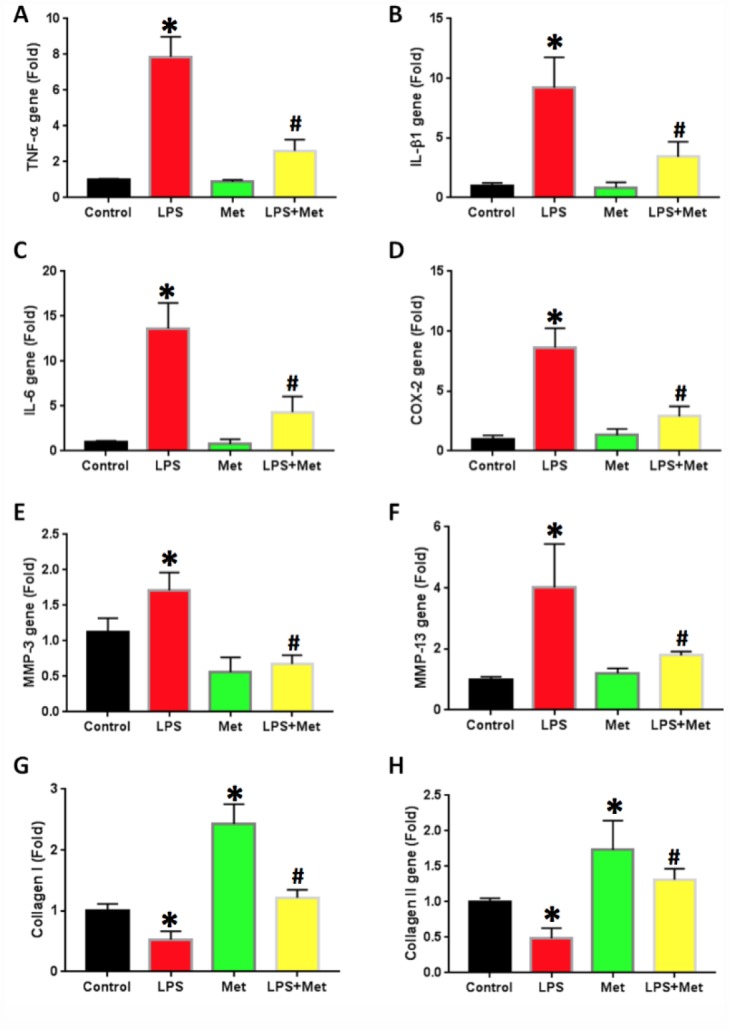
**Metformin inhibited the inflammatory gene expression in rabbit AF cells induced by LPS.** (**A**) TNF-a gene expression. (**B**) IL-b1 gene expression. (**C**) COX-2 gene expression. (**D**) IL-6 gene expression. (**E**) MMP-3 gene expression. (**F**) MMP-13 gene expression. (**G**) Collagen I gene expression. (**H**) Collagen II gene expression. LPS increased the expression of all tested inflammatory-related genes and MMPs, but decreased the levels of collagen type I and type II. The LPS effect was inhibited by metformin. *p<0.05 to the control group. ^#^p<0.05 compared to the cells treated with LPS.

The expression of the catabolic genes in AF stem cells was also studied. AF stem cells treated with LPS indicated significant upregulation of catabolic gene expression including *MMP-3* (Figure 7E) and *MMP-13* (Figure 7F) compared with those noted in the untreated control samples. Moreover, metformin treatment significantly decreased the expression levels of these genes in AF stem cells (Figure 7). The gene expression levels of collagen type I (*Col-I*) were further decreased in LPS-treated AF cells, while they were increased by metformin treatment (Figure 7G). Similarly, collagen type II (*Col-II*) gene expression levels were also decreased in AF cells treated with LPS (Figure 7H), while they were increased in metformin-treated cells (Figure 7H).

**Figure 8:**
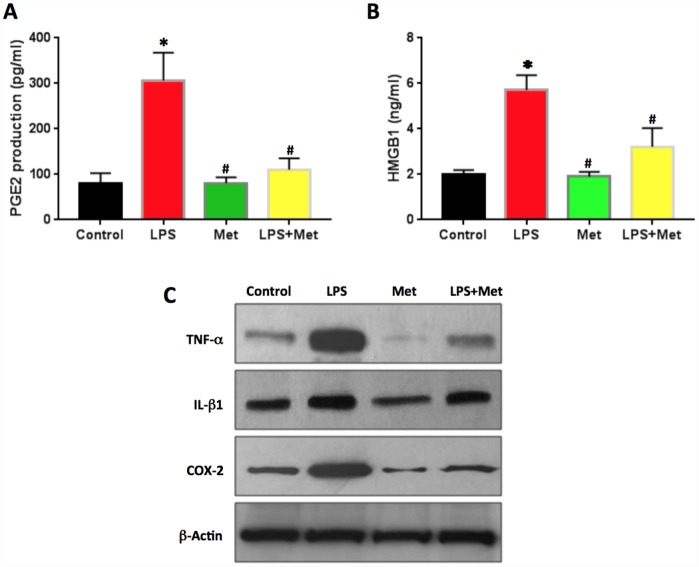
**Anti-inflammatory effect of metformin on rabbit AF cells induced by LPS.** (**A**) PGE2 production in the medium of rabbit AF cells tested by ELISA. (**B**) HMGB1 concentration in the medium of rabbit AF cells tested by ELISA. (**C**) Inflammatory-related marker protein expression in rabbit AF cells tested by western blot. ELISA results indicated that LPS induced the inflammation in rabbit AF cells as evidenced by PGE2 production and HMGB1 concentration found in the medium of rabbit AF cells cultured with LPS. Effect was inhibited by metformin. *p<0.05 to the control group. ^#^p<0.05 compared to the cells treated with LPS.

PGE2 production in LPS-treated rabbit AF cells was two times higher than that of the control cells (Figure 8A). Metformin decreased PGE2 production significantly in rabbit AF cells (Figure 8A).

The concentration of HMGB1 in the culture medium of rabbit AF cells was also increased by LPS treatment (Figure 8B). Metformin decreased the levels of HMGB1 in the medium of the cells treated with LPS (Figure 8B). Western blot analysis highlighted that the protein levels of TNF-α, IL-β1 and COX-2 were enhanced by LPS treatment and decreased by metformin treatment (Figure 8C).

## DISCUSSION

Low back pain is the most common musculoskeletal problem encountered worldwide and causes significant socio-economic burden. Intervertebral disc degeneration (IVDD) is one of the major causes of low back pain [2]. However, the mechanism of IVDD is still unclear [5]. It has been reported that induction of cell apoptosis, excessive pro-inflammatory cytokine release (cytokine storm) and increased matrix catabolism play a key role in the pathogenesis of IVDD [6]. However, the effective treatment options are limited [6].

In order to study the mechanisms of IVDD and identify an efficient approach for low back pain treatment, rabbit AF stem cells were treated with metformin and LPS. The results indicated that LPS induced HMGB1 release from the nuclei of rabbit AF stem cells and caused cell senescence. The production of PGE2 and HMGB1 was also increased in the medium of AF cells treated with LPS. The expression of certain inflammation-related genes, such as *IL-β1*, *IL-6* and *TNF-α* was increased, whereas the expression levels of specific anabolic genes, such as *Col I* and *Col-II* were decreased in LPS-treated AFSCs. The effects of LPS were inhibited by metformin. The findings indicated that metformin exhibited anti-inflammatory effects by blocking HMGB1 translocation and by inhibiting the catabolic products of AFSCs.

Inflammation and cell senescence play critical roles in the pathogenesis of IVDD. The intervertebral disc consists of the three following components: a central nucleus pulposus (NP), a peripheral annulus fibrosus (AF) and two vertebral endplates. As one of the main types of resident cells in the disc tissues, the proliferation of AFSCs affects disc regeneration and degeneration. The present study indicated that following treatment of AFSCs with LPS, HMGB1 was released from the nuclei into the cytoplasm, resulting in increased inflammatory cytokine release. It has been shown that the upregulation of inflammatory cytokines, notably tumor necrosis factor-α (TNF-α) and interleukin-1β (IL-1β) contributes to the progression of IVDD by inducing aggrecan degradation [25]. The results of the present study indicated that HMGB1 cellular translocation might be the key cause for the development of IVDD.

Cellular senescence contributes to age-associated tissue dysfunction, reduced regenerative capacity and disease [26]. Senescent cells are found in various types of organs and tissues. Senescence is closely associated with aging. Previous studies indicated that cellular senescence caused fundamental changes in the disc cells, ultimately leading to IVDD development [27]. HMGB1 has been considered a central mediator of senescent phenotypes [28]. The loss of nuclear HMGB1 expression is considered a marker of senescent cells [29]. These findings were further confirmed by the results reported in the present study. Prior to treatment, the morphology of the normal AFSCs exhibited a spindle-like shape and HMGB1 was localized in the cell nucleus. However, following LPS treatment, AFSCs exhibited epithelial-like shape and HMGB1 expression was not present in the cell nucleus. Furthermore, LPS-treated AFSCs were positively stained with SA-β-gal, indicating that these cells were senescent cells. A previous *in vivo* study demonstrated that inhibition of cell apoptosis attenuated the degenerative progression of the rat intervertebral disc [30].

Senescent cells accumulate with age and are detrimental for tissue structure and function. Therefore, aging is a risk factor for several diseases [29]. The present study indicated that the senescent phenotype induced by LPS could be inhibited by metformin. The addition of metformin to LPS-treated AFSCs blocked the HMGB1 release as demonstrated by the high percentage of cells retaining the HMGB1 protein in their nuclei and the limited number of cells exhibiting senescent markers. Moreover, decreased production of PGE2 and HMGB1 was noted in the medium and the expression levels of the pro-inflammatory cytokines (IL-1b, IL-6, TNF-α) and of the metalloproteinases (MMPs) were decreased. These findings were also supported by a previous *in vivo* study [31].

Metformin has been widely used for the treatment of type 2 diabetes. Recently, metformin was found to possess an anti-inflammatory effect. However, the precise mechanism of its anti-inflammatory effect remains to be clarified [32]. Recent studies indicated that metformin stimulated cell autophagy in various tissues and organs, such as the brain [33, 34], kidney [35], and heart cells [36], and the intervertebral disc NP cells [31]. An additional study demonstrated that metformin was an inhibitor of HMGB1 and that it could directly bind to the alarmin HMGB1 to inhibit the proinflammmatory activity of HMGB1 [32]. The present study further demonstrated that metformin blocked HMGB1 release from the cell nucleus to the cytoplasm. It concomitantly resulted in a partial decrease of the senescence-associated β-gal activity and to a dramatic decreased of the expression levels of TNF-α, IL-1β, IL-6, MMP-3, and MMP-13 that were induced by LPS in AF stem cells. The findings indicated that metformin may represent a new therapeutic drug for preventing and treating disc inflammation and degenerative disc disease formation including IVDD.

## MATERIALS AND METHODS

### Isolation of stem cells from AF tissues

The AF tissue samples were obtained from the lumbar spines of three New Zealand white rabbits (5 months old). The protocol for the use of the rabbits was approved by IACUC of the Tongji University, China. The AF tissues were cut into small pieces and digested with phosphate buffer solution containing 0.4% pronase (100 mg tissue/ml enzyme) at 37°C for 1 h. Subsequently, the samples were incubated with phosphate buffered 0.04% collagenase P solution (100 mg tissue/ml enzyme) overnight. The undigested tissue pieces were removed by a 70 μm filter, the supernatant was centrifuged at 500 g for 10 min and the pellets were washed three times with PBS. A single-cell suspension was obtained by re-suspending the cell pellets in culture medium consisting of F-12 medium (Lonza, Walkersville, MD) supplemented with 20% fetal bovine serum (FBS; Atlanta Biological, Lawrenceville, GA), 100 U/ml penicillin and 100 μg/ml streptomycin (Atlanta Biologicals, Lawrenceville, GA). The cells were cultured in T25 flasks at a density of 2 × 10^5^/flask. Following 10 days of culture, the stem cells that were isolated from AF tissues (AFSCs) formed colonies on the surface of the flask.

### Purification and identification of AFSCs

For stem cell purification, individual cell colonies were collected by local application of trypsin under microscopic visualization and transferred to the individual wells of 6-well plates for further culture using published previously published method [37].

To identify the stem cells, the purified AFSCs at passage 2 were cultured in 12-well plates (3 × 10^4^ cells/well) for 5 days. The cells were fixed at room temperature with phosphate buffer containing 4% paraformaldehyde for 30 min. The fixed cells were directly reacted with mouse anti-stage-specific embryonic antigen-4 (SSEA-4; 1:500, Cat. #414000, Invitrogen, Carlsbas, CA) at 4°C overnight. For octamer-binding transcription factor-4 (Oct-4) and nucleostemin (NS) staining, the fixed cells were further treated with 0.1% triton X-100 for 30 min and subsequently incubated either with mouse anti-Oct-4 antibody (1:350, Cat. #MAB4401, Millipore, Temecula, CA), or goat anti-nucleostemin antibody (1:500, Cat. # GT15050, Neuromics, Edina, MN) at 4°C overnight. Following washing of the cells three times with PBS, cyanine 3 (Cy3)-conjugated goat anti-mouse immunoglobulin G (IgG) secondary antibody (1:500, Cat. # A10521, Invitrogen, Carlsbas, CA) was used for SSEA-4 and Oct-4 determination. Furthermore, Cy-3 conjugated donkey anti-goat IgG antibody (1:500, Cat. # AP180C, Millipore, Temecula, CA) was used for nucleostemin testing. The cells were also counterstained with Hoechst 33342 (1 μg/ml, Cat. #33270, Sigma, St Louis).

### The effect of metformin on cell proliferation

To determine the effect of metformin on cell proliferation, the AFSCs at passage 2 (1.5 × 10^4^/well) were cultured in a 12-well plate with culture medium overnight. The following day, various concentrations of metformin (0, 1, 5, 10 mM) were added into the culture medium and the culture was performed for an additional 5 days. The morphology of the cells was observed by a microscope. Cell proliferation was determined by estimating the population doubling time, (PDT) which was defined as the total culture time divided by the number of generations described as reported in a previous study.^28^ The number of generations was expressed as log_2_Nc/No, where No was the population of the cells seeded initially, and Nc the population at confluence [37].

### The effect of LPS on morphological changes and HMGB1 release of AFSCs

The effect of LPS on AFSCs was determined by morphological changes. The AFSCs at passage 2 (1.5 × 10^4^/well) were cultured in a 12-well plate with culture medium overnight. The following day, various concentrations of LPS (0, 10, 50, 100 ng/ml) were added into the culture medium and the culture was carried out for an additional 5 days. The morphology of the cells was observed by microscopy. The number of the cells with different shape was counted manually and expressed as percentage.

HMGB1 expression in AFSCs was determined by immunostaining. Briefly, the cells were cultured with various concentrations of LPS and fixed at room temperature with 4% phosphate-buffered paraformaldehyde for 30 min. The fixed cells were further treated with 0.1% triton X-100 for 30 min and washed with PBS for three times. Subsequently, the fixed cells were incubated with mouse anti-HMGB1 antibody (1:300, antibodies online.com, Cat. #ABIN1176834) at room temperature for 2 h. Following washing for three times with PBS, the cells were incubated with cy3-conjugated goat anti-mouse IgG secondary antibody (1:500, Cat. # A10521, Invitrogen, Carlsbas, CA) at room temperature for 1 h. The total number of the cells was determined by counterstaining with Hoechst 33342 (1 μg/ml, Cat. #33270, Sigma, St Louis).

### The inhibitory effect of metformin on HMGB1 release and cell senescence

To determine the inhibitory effect of metformin on the LPS-induced nuclear translocation of HMGB1, AFSCs at passage 2 (1.5 × 10^4^/well) were cultured overnight in a 12-well plate with culture medium. The following day, the cells were treated with four different conditions. Group-1 contained the cells that were cultured with culture medium only (Control), group-2 contained the cells that were cultured in culture medium with 100 ng/ml LPS (LPS), group-3 included the cells that were cultured in culture medium with 1 mM of metformin (Met) and group 4 contained the cells that were cultured in culture medium with 100 ng/ml LPS and 1 mM metformin (LPS+Met). Following 5 days of culture, the medium from each group was collected for the determination of the prostaglandin E2 (PGE2) production and for the measurement of HMGB1 concentration using the commercial kits (Cat. #514010, for PGE2 determination, Cayman Chemical and Cat. #ST51011 for HMGB1 determination, Ann Arbor, MI; IBL International GmbH, Hamburg, Germany) according to the manufacturer’s protocols. The HMGB1 expression in AFSCs was also determined by immunostaining as described by the aforementioned protocol. The cell senescence in AFSCs was determined by the senescence β-galactosidase staining kit (Cell Signaling Technology, Cat. #9860, Danvers, MA) according to the manufacturer’s instructions.

### Quantitative real-time RT-PCR for gene analysis

The metformin and LPS effects on AFSCs were further assessed by qRT-PCR. Following 5 days of culture with four different conditions, RNA was extracted from the cells using an RNeasy Mini Kit (Qiagen, Cat. #74104, Pudong, Shanghai, China). The gene expression was tested using the Qiangen QuantiTeact SYBR Green RT-PCR Kit (Qiagen, Cat. #204243, Pudong, Shanghai, China) in a Real Time PCR System (Step One Plus, AB Applied Biosystems). Rabbit specific primers were used for the detection of collagen type I (*Col-I*), collagen type II (*Col-II*), *MMP-3*, *MMP-13*, *IL-6*, *IL-β1*, *COX-2*, and *TNF-α* genes. Glyceraldehyde-3-phosphate dehydrogenase (*GAPDH*) was used as an internal control. The forward and reverse primer sequences were designed according to previous studies and are listed in Table 1 [38–40]. Following an initial denaturation step for 10 min at 95°C, PCR was performed for 80 cycles. Each cycle consisted of the following steps: denaturation for 50 sec at 95°C, followed by annealing for 50 sec at 58°C and extension for 40 sec at 72°C. The PCR reaction was terminated following a 10-min extension at 70°C. At least three independent experiments were performed to obtain the relative expression levels of each gene.

**Table 1:** Rabbit primer sequences used for qRT-PCR analysis.

**Gene**	**Forward**	**Reverse**	**Reference**
TNF-a	5′-CTGCACTTCAGGGTGATCG-3′	5′-CTA CGT GGG CTA GAG GCT TG-3′	39
IL-1b	5′-TTG AAG AAG AAC CCG TCC TCT G-3′	5′-CTC ATA CGT GCC AGA CAA CAC C-3′	39
IL-6	5′-CTA CCG CTT TCC CCA CTT CAG-3′	5′-TCC TCA GCT CCT TGA TGG TCT C-3′	39
COX-2	5′-CAC GCA GGT GGA GAT GAT CTA C-3′	5′ -ACT TCC TGG CCC ACA GCA AAC T-3′	38
MMP-3	5′-ACA CCG GAT CTG CCA AGA GA-3′	5′-CTG GAG AAC GTG AGT GGA GTC A-3′	38
MMP-13	5′-CAT GCC AAC AAA TTC CCT GCT GTG GT-3′	5′-TCT CCT CCC TGC ACC TCC AGA TTT-3′	38
Collagen I	5′-CAA TCA CGC CTC TCA GAA CA-3′	5′-TCG GCA ACA AGT TCA ACA TC-3′	40
Collagen II	5′-CAA CAA CCA GAT CGA GAG CA-3′	5′-CCA GTA GTC ACC GCT CTT CC-3′	40
GAPDH	5′-TGA CGA CAT CAA GAA GGT GGT G-3′	5′-GAA GGT GGA GGA GTG GGT GTC-3′	39

### Western blot analysis

The effects of metformin and LPS on the expression of specific proteins of AFSCs were also assessed by western blot analysis. Following 5 days of culture with four different conditions, the protein was extracted from the cells using a lysis buffer (Thermo Scientific, Cat. #78501, Pittsburgh, PA). In order to ensure equal loading, the total protein concentration in each sample was measured by a BCA protein assay kit (Thermo Scientific, Cat. #23225, Pittsburgh, PA). A total of 30 μg protein was separated in 10% SDS gels by PAGE prior to their transfer to PVDF membranes (Bio-Rad, Hercules, CA). The blots were blocked using 5% Non-Fat dry milk (Bio-Rad, Hercules, CA) at room temperature for 1 h and subsequently incubated at 4°C overnight with primary antibodies against TNF-α (Cell Signaling, 1:1,000), IL-β1 (Cell Signaling, 1:1,000), COX-2 (Cell Signaling, 1:1,000) and β-actin (Abcam, 1:10,000). The following day, the blots were washed three times with PBS-T buffer and incubated with the corresponding secondary antibodies (LI-COR Biosciences, 1:15,000) for 1 h at room temperature. Following another three washes with PBS-T, the blots were subjected to the LiCoR Odyssey imager (LI-COR Biosciences, Lincoln, NE) for visualization of the protein bands, and semi-quantification was performed using the software of the LiCoR Odyssey imager.

### Statistical analysis

All experiments were performed in three replicates. The data are presented as mean ± SD.

One-way analysis of variance (ANOVA), followed by Fisher’s predicted least-square difference (PLSD) for multiple comparisons, or two tailed student t-test wherever applicable, were used for statistical analysis. The differences between the two groups were considered significant when the *p*-value was less than 0.05.
